# Post-Translocation Establishment of the Endemic Cyprinid *Squalidus multimaculatus* Under Favorable Biogeochemical Conditions and Regional Winter Warming

**DOI:** 10.3390/biology15141140

**Published:** 2026-07-13

**Authors:** Sun Kyeong Choi, Seul Yi, Samuel Praveen, Young Baek Son, Seonggil Go

**Affiliations:** 1 Tropical & Subtropical Research Center, Korea Institute of Ocean Science and Technology, Jeju 63349, Republic of Korea; choisk@kiost.ac.kr (S.K.C.); dearrei0711@gmail.com (S.Y.); samuelpraveen@kiost.ac.kr (S.P.); 2 Ocean Science, KIOST School, University of Science and Technology, Daejeon 34113, Republic of Korea

**Keywords:** biogeochemical suitability, critical cold stress, thermal window, population structure, demographic shift, climate change, growth curve, ecosystem evolution

## Abstract

Human-mediated species translocations are increasingly interacting with global climate change, yet empirical insights into how introduced populations achieve long-term demographic stability in new frontiers remain limited. This study systematically investigates the post-translocation establishment and multi-generational persistence of the endemic cyprinid *Squalidus multimaculatus* at its newly identified northern distribution limit in Goseong, Republic of Korea, following historical human-mediated introduction. Utilizing nationwide multi-decadal occurrence records, we mapped the species’ spatio-temporal dynamics across three temporal phases (*T*_0_, *T*_1_, and *T*_2_). Long-term biogeochemical water quality indices and thermal regimes were compared between the native baseline (Yeongdeok) and Goseong. Furthermore, demographic shifts and growth trajectories were evaluated using 676 field-sampled specimens through length–weight relationships, condition factor (*K_F_*) analysis, age structure mixture modeling, and the von Bertalanffy growth function (VBGF). Our results indicate that the Goseong habitat provides generally favorable biogeochemical conditions characterized by low nutrient loading and high dissolved oxygen stability. Crucial to this transition, a distinct winter warming shift expanded the available thermal window, reducing the frequency of extreme winter cold-stress events (≤2.0 °C) from 40.0% to 6.9%. Concurrently, the post-translocation population exhibited successful demographic stability, characterized by a self-sustaining age structure (ages 0+ to 4+), an inferred spawning window during June–July, and a growth trajectory (*L*_∞_ = 95.69 mm) broadly comparable to native benchmarks. These findings establish a reliable empirical baseline framework linking human-mediated introductions with long-term environmental transitions. Globally, this case study suggests that post-translocation success depends on the interplay between introduction pathways and climate suitability, offering key insights into population survival and ecosystem evolution under global change.

## Simple Summary

Climate change poses an escalating threat to freshwater ecosystems worldwide, particularly affecting endemic species with restricted geographic ranges and narrow environmental tolerances. This study investigates the post-translocation establishment and multi-generational persistence of *Squalidus multimaculatus*, a small freshwater fish endemic to the Korean Peninsula, at its newly identified northernmost population in Goseong, Republic of Korea, following historical human-mediated introduction. Our findings reveal that severe historical winter temperatures in Goseong once acted as a significant thermal barrier; recent regional climate warming has dramatically reduced the frequency of critical winter cold-stress events from 40.0% to 6.9%. Supported by favorable biogeochemical conditions featuring high dissolved oxygen and low phosphorus loading, this translocated cohort has successfully established a self-sustaining population with a stable multi-generational age structure (Ages 0+ to 4+) and robust growth vitality comparable to its native benchmarks. By combining long-term climate tracking with empirical population traits, this study provides vital scientific baselines to support proactive managed translocation and conservation strategies for climate-sensitive endemic species under global change.

## 1. Introduction

Climate change is causing rapid shifts in ecosystems worldwide, altering species distributions, population dynamics, and community structures across various spatio-temporal scales, while driving numerous species toward extinction [1,2]. In this context, profound fluctuations in biodiversity have been observed within freshwater ecosystems, whose biota are exceptionally vulnerable to climatic alterations [3,4]. Due to the inherent isolation of river networks, freshwater organisms face severe physical constraints when attempting to migrate actively to track changing thermal niches [4,5]. Beyond thermal niche availability, the structural complexity and functional integrity of the surrounding ecosystem play a deterministic role in regulating fish population demographics; globally, ecosystem shifts—such as the degradation of riparian macrophyte beds in European river systems [6] or the loss of woody debris architecture in North American streams [7]—directly impair fish abundance by dismantling critical spawning substrates and juvenile foraging refugia [8]. Furthermore, because these systems are closely intertwined with areas of high human density, they are directly exposed to anthropogenic stressors, escalating the risk of rapid, climate-driven extinctions [9,10]. As of May 2026, approximately 19,000 valid species of freshwater fish have been identified globally, accounting for more than half of all known fish species [11]. Unfortunately, one-quarter of these freshwater fish are currently threatened with extinction, and the modern extinction rate since 1851 has been reported to be over 100 times higher than the natural background rate [12,13].

When localized habitats undergo environmental shifts, organisms must disperse to locate alternative thermal and ecological refugia suitable for survival [14,15]. Climate drivers, particularly air and water temperatures, exert primary control over fish distributions, in tandem with physical and biogeochemical variables such as river morphology, dissolved oxygen, and nutrient loading [8,10]. Because the fragmented nature of river systems restricts the autonomous migration of freshwater fish, populations tracking thermally optimal conditions tend to shift upstream toward higher altitudes or poleward (northward), or establish new populations via inadvertent anthropogenic translocations [5,10,16]. Consequently, predictive models suggest that freshwater fish assemblages will undergo drastic spatial reconfigurations under various climate change scenarios [17,18]. However, empirical data remain scarce on the mechanisms through which expanding species successfully colonize new frontiers, particularly concerning the interactions between modified habitat characteristics and population-level growth responses. In particular, this gap is even more critical for endemic species, which often exhibit narrow environmental tolerances and smaller population sizes, rendering them disproportionately susceptible to global warming [19].

A total of 2370 endemic species inhabit the Republic of Korea, including 72 fish species, most of which are freshwater species according to the National Institute of Biological Resources database (NIBR, online database, accessed on 30 May 2026). Within the cyprinid genus *Squalidus*, 18 valid species are recognized worldwide; among these, four are distributed in the Republic of Korea and are entirely endemic to the Korean peninsula: *Squalidus gracilis majimae*, *Squalidus japonicus coreanus*, *Squalidus chankaensis tsuchigae*, and *Squalidus multimaculatus* [20]. While the former three species are widely distributed across large river systems draining into the western and southern coasts of Korea, *S. multimaculatus* is uniquely restricted to independent streams draining into the East Sea. First described as a new species in 1984 from the Yeongdeok-Osip Stream [21], Jeon et al. (2018) demonstrated through genetic structure analysis that *S. multimaculatus* split from *S. g. majimae* approximately 1 million years ago due to tectonic fault movements that isolated the eastern river systems [22]. Historically, *S. multimaculatus* was predominantly confined to the coastal streams of the southeastern peninsula, south of 36° N [23]. However, recent surveys have captured a notable northward range expansion extending up to 38° N [24,25], a phenomenon hypothesized to be driven by anthropogenic pathways [23,26].

Previous research on *S. multimaculatus* has mainly focused on community-level species diversity surveys [24,25] or molecular investigations aimed at clarifying phylogenetic lineages and evolutionary genetics [22,26], comparative morphology [27], and basic life-history traits such as age structure and sexual maturation [28,29]. Despite these contributions, systematic evaluations of the species’ macro- and micro-habitat requirements remain limited, and detailed analyses of its population growth dynamics in newly colonized habitats are currently lacking. Given that endemic species are confined to specific geographic areas, understanding their ecological shifts during rapid environmental transitions is critical for resource management and biodiversity conservation [24,28]. Information on the range dynamics of such climate-sensitive endemics remains highly sparse, and studies integrating long-term habitat variations with empirical biological traits are even rarer.

To address these critical gaps, the primary objective of this study is to systematically elucidate the environmental conditions and population-level traits associated with the post-translocation establishment of the endemic cyprinid *S. multimaculatus*. Given the strict hydrological isolation of independent coastal streams on the eastern slope of the Korean Peninsula, we assume an anthropogenic translocation pathway for the initial founder population based on historical regional context [24,26,30]. On this premise, we test the primary hypothesis that regional climate-driven thermal homogenization dismantled historical winter cold-stress barriers, thereby unlocking the northern thermal window and enabling long-term, multi-generational persistence under favorable biogeochemical conditions. Ultimately, this study establishes a streamlined, data-driven framework combining regional shifting dynamics with empirical population traits to support the proactive management and conservation of climate-sensitive endemic freshwater fish.

## 2. Materials and Methods

### 2.1. Study Area and Target Species

**Figure 1:**
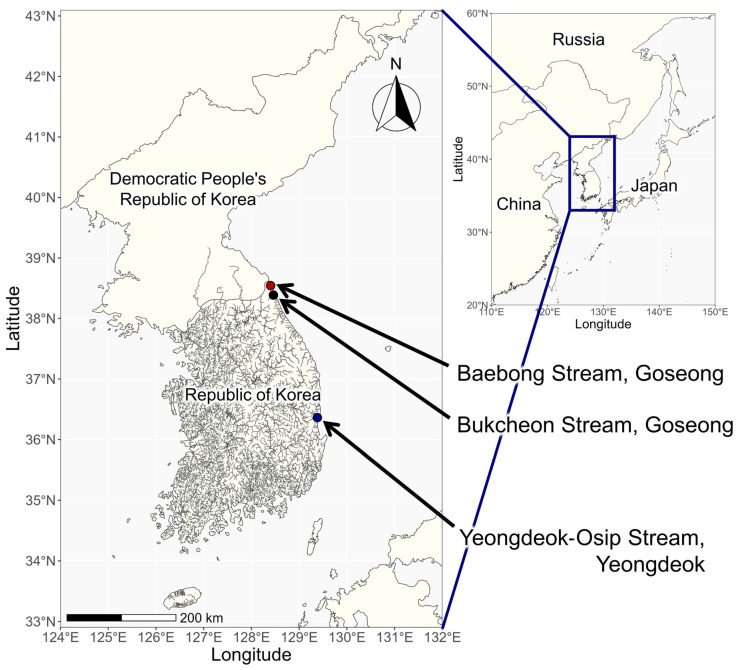
Map of the study area on the Korean Peninsula. The blue circle represents the original native habitat of *Squalidus multimaculatus* (the Yeongdeok-Osip Stream in Yeongdeok). The red circle indicates the newly colonized Baebong Stream in Goseong, where biological sampling was conducted. In contrast, the black circle denotes the adjacent Bukcheon Stream, which served as a regional proxy for analyzing the environmental and thermal baselines. The river network layer was obtained from the Water Resources Management Information System (https://wamis.go.kr, accessed on 30 May 2026).

We selected *Squalidus multimaculatus*, a small freshwater fish endemic to the Korean Peninsula, as the target species for this study. While its original native habitats are primarily located in the southeastern streams of the Republic of Korea (e.g., Yeongdeok), the primary study area for our biological investigation was designated as the Baebong Stream in Goseong, South Korea (Figure 1). Due to the absence of a national long-term water quality monitoring station within the Baebong Stream, environmental and thermal baseline data were obtained from the hydrologically and geographically adjacent Bukcheon Stream (approximately 17 km apart at their river mouths), which served as a regional environmental proxy. This spatial proxy approach is supported by three parallel congruences summarized in Table S1. (1) Macroclimatic proximity: both streams share highly similar atmospheric forcing and thermodynamic regimes within the same 0.25° ERA5 climate grid cell, empirically verified by strong daily thermal synchronization between the Korea Meteorological Administration (KMA) automated weather stations (*r*^2^ = 0.995, RMSE = 0.72 °C), as well as highly accurate grid-to-ground correspondences for the ERA5 dataset against both the Bukcheon (*r*^2^ = 0.972) and Baebong (*r*^2^ = 0.966) ground observations (Figure S1); (2) microhabitat congruence: the lower reaches of both catchments exhibit identical Bc-type river morphologies with heterogeneous mixed substrates (pebble-dominant [Pebble > Sand = Mud = Cobble] in Baebong and sand-dominant [Sand > Gravel > Mud > Pebble = Cobble] in Bukcheon) that functionally satisfy the benthic substrate-sifting and sheltering behaviors of Gobioninae [24,30,31]; and (3) regulatory coherence: both catchments are strictly protected as municipal water sources (Hyeonnae and Ganseong Water Supply Protection Areas, respectively), restricting anthropogenic loading and ensuring synchronous, favorable water quality baselines (https://www.gwgs.go.kr/kor/sub02_070201.do, accessed on 23 June 2026). Baebong Stream currently represents the newly identified northernmost limit of the species’ distribution, providing a favorable natural laboratory to study early-stage population establishment and ecological adaptation. The primary dataset regarding the sampling and size distribution of *S. multimaculatus* was obtained from the monitoring records originally presented in Yi (2020) [23].

### 2.2. Occurrence Data and Distribution Mapping

To trace the spatio-temporal distributional patterns of *S. multimaculatus* across the Korean Peninsula, we compiled extensive nationwide occurrence records from five major ecological databases: (1) the National Ecosystem Survey and (2) the Wetland Information System, both provided by the National Institute of Ecology (https://www.nie-ecobank.kr, accessed on 30 May 2026); (3) the Aquatic Ecosystem Health Assessment conducted by the National Institute of Environmental Research (https://water.nier.go.kr, accessed on 30 May 2026); (4) the Biodiversity of the Korean Peninsula database managed by the National Institute of Biological Resources (https://species.nibr.go.kr/, accessed on 30 May 2026); and (5) the Global Biodiversity Information Facility (GBIF Occurrence Download. Available online: https://www.gbif.org, accessed on 30 May 2026). These public database records were supplemented with verified occurrence data extracted from regional literature for the Baebong [23,24,25] and Bukcheon [30] Streams in Goseong. To ensure data reliability, the compiled datasets were rigorously reviewed for quality control; geographical outliers that were distinctly isolated from established distribution ranges and thus strongly suspected to be recording, typographical, or misidentification errors were excluded from the subsequent analyses. We filtered the compiled occurrence coordinates and categorized them into three distinct temporal periods—pre-2005 (*T*_0_), 2006–2015 (*T*_1_), and post-2016 (*T*_2_)—to spatially visualize and map the historical northward shift in the distribution range of the species. Spatial distribution mapping of the occurrence data was conducted using the “rnaturalearthdata” package (version 1.0.0) in R [32]. While these consolidated multi-agency records inherently reflect historical variations in national sampling intensity and detection probabilities rather than standardized continuous monitoring, they provide a reliable geographic indicator to trace macro-scale distributional boundaries.

### 2.3. Environmental Data Acquisition and Analysis

#### 2.3.1. Biogeochemical, Physical, and Microbiological Water Quality

To evaluate the fundamental habitat suitability and identify environmental conditions associated with post-translocation establishment, we compared comprehensive water quality baselines between the native (Yeongdeok) and colonized (Goseong) habitats. We obtained the monthly data on 8 environmental variables from January 1997 to December 2025 from the Water Environment Information System of the Republic of Korea (https://water.nier.go.kr, accessed on 30 May 2026). Specifically, water quality datasets from the nearby Bukcheon Stream were utilized as a spatial proxy to represent the macro-environmental and biogeochemical baselines of the Goseong coastal stream ecosystem. We then established the historical environmental conditions of the native Yeongdeok habitat (pre-2005) as the ecological baseline (hereafter Y-*T*_0_), and compared this baseline against the spatio-temporal environmental shifts in Goseong across three distinct phases corresponding to the species’ distribution history: (1) pre-colonization (pre-2005; hereafter G-*T*_0_), (2) early emergence (2006–2015; hereafter G-*T*_1_), and (3) recent establishment (post-2016; hereafter G-*T*_2_). For this analysis, we calculated 8 annual mean parameters encompassing physical (suspended solids, SS), biogeochemical (dissolved oxygen, DO; biochemical oxygen demand, BOD; chemical oxygen demand, COD; total nitrogen, TN; total phosphorus, TP), and microbiological (total coliforms, Total_Coli; fecal coliforms, Fecal_Coli) conditions.

Due to the non-normal distribution typical of long-term environmental monitoring data, we employed the non-parametric Kruskal–Wallis rank-sum test to ascertain statistically significant differences across the four temporal groups (Y-*T*_0_, G-*T*_0_, G-*T*_1_, and G-*T*_2_) [33]. Additionally, to establish a robust environmental baseline, we evaluated and classified the 8 environmental parameters according to the statutory water quality grades (Grades Ia to VI) stipulated by the Framework Act on Environmental Policy of the Republic of Korea (Table S2; https://www.law.go.kr, accessed on 30 May 2026). Furthermore, to account for underlying long-term monotonic trends and seasonality across the 29-year tracking period (1997–2025), formal Seasonal Mann–Kendall trend tests accompanied by Sen’s slope estimators were conducted for all monthly water quality variables. To preserve the empirical integrity of the background baseline without introducing artificial imputation artifacts, missing monthly observations were handled explicitly via the pairwise deletion algorithm implemented in the ‘EnvStats’ package (version 3.1.0) in R [34].

#### 2.3.2. Thermal Dynamics and Critical Cold Stress

To evaluate climatic shifts and their influence on the aquatic habitat, we analyzed the spatio-temporal dynamics of both air temperature (Atemp) and water temperature (Wtemp). Daily 2 m air temperature data spanning 86 years (1940–2025) were acquired from the European Centre for Medium-Range Weather Forecasts Reanalysis 5 (ERA5) product (0.25° resolution; https://climate.copernicus.eu/climate-data-store, accessed on 30 May 2026) for the Yeongdeok and Goseong regions. To ensure the reliability of the reanalysis data for localized thermal regime assessment, the extracted ERA5 midday temperatures were cross-validated against empirical ground observations from the nearest KMA station in Goseong (2016–2025), which demonstrated highly consistent temporal variability (Figure S1). Concurrently, the corresponding water temperature data were retrieved from the Water Environment Information System of the Republic of Korea (https://water.nier.go.kr, accessed on 30 May 2026), consistent with the aforementioned water quality monitoring framework. The historical baseline was defined distinctly for each thermal variable based on historical data availability. To capture long-term macroclimatic trends, the air temperature baseline was established from 1940 to 2005 (hereafter denoted as *T*_0_*). In contrast, due to the tracking constraints of the monitoring network, the water temperature baseline was set from 1997 to 2005 (consistent with the water quality baseline, hereafter *T*_0_). Using these parameter-specific baselines (Y-*T*_0_* and G-*T*_0_* for air temperature; Y-*T*_0_ and G-*T*_0_ for water temperature), we compared the thermal shifts against the subsequent phases (G-*T*_1_ and G-*T*_2_).

Because the raw air temperature data consisted of daily estimates, we initially aggregated the records into monthly means to align the temporal resolution with the water temperature data. Subsequently, for each spatio-temporal group, we calculated 3 specific annual indices for both parameters: the annual mean temperature (Mean_Atemp and Mean_Wtemp), the average of the minimum monthly temperatures per year (Min_Atemp and Min_Wtemp), and the average of the maximum monthly temperatures per year (Max_Atemp and Max_Wtemp), yielding a total of 6 comprehensive thermal datasets.

Following the same statistical approach as in the environmental variable analysis, we utilized the non-parametric Kruskal–Wallis rank-sum test [33]. When significant differences were detected (*p* < 0.05), we subsequently applied Dunn’s post hoc test with Bonferroni correction to identify the precise periods of thermal transition [35]. Additionally, to evaluate continuous macro-climatic trajectories without serial autocorrelation bias, standard Mann–Kendall trend tests and Sen’s slope estimators (‘EnvStats’ package) were applied to the annual aggregated water temperature indices (Mean_Wtemp, Min_Wtemp, and Max_Wtemp) over the 1997–2025 monitoring period [34]. Furthermore, we calculated the root-mean-square error (RMSE) between air and water temperatures to quantify thermal coupling efficiency [36], thereby evaluating how closely the aquatic thermal regime tracks atmospheric climate change. Finally, to assess the alleviation of physiological barriers for the overwintering survival in *S. multimaculatus*, we quantified the occurrence ratio of critical cold-stress events. These events were defined as periods when the water temperature dropped to ≤2.0 °C, a conservative working threshold established based on the previous literature reporting that the critical lower thermal tolerance for various temperate cyprinid species typically ranges from approximately 2.0 °C to 6.0 °C [37]. To address the uncertainty arising from the lack of species-specific thermal tolerance data for *S. multimaculatus*, we explicitly treated this threshold as an ecological assumption. Consequently, we performed a sensitivity analysis by varying the threshold by ±1.0 °C (i.e., quantifying the occurrence ratios at ≤1.0 °C and ≤3.0 °C) to evaluate the sensitivity of the winter warming interpretation to threshold choice.

### 2.4. Biological Data Collection and Analysis

#### 2.4.1. Field Sampling

Field sampling was conducted in the Baebong Stream, Goseong, Republic of Korea, targeting the newly identified northernmost distribution limit of *S. multimaculatus* [23]. The field surveys were carried out from April to September 2014, during which a total of 676 individuals were successfully collected. Due to the single-year and single-season nature of this sampling, we explicitly acknowledge that this dataset inherently limits direct inferences regarding continuous, inter-annual population stability. To confirm the presence of *S. multimaculatus* prior to field collection, an underwater video recording system (GoPro HERO4 Silver; GoPro Inc., San Mateo, CA, USA) was deployed at each survey point for 1 h, and active sampling was initiated exclusively at micro-habitats where the species’ localized occurrence was visually verified. To accommodate spatial variations in channel dimensions and river morphology within the downstream reach, sampling operations followed a structured gear-selection framework based on three primary criteria (fully summarized in Table S3): (1) microhabitat classification, distinguishing target zones into deep pools or shallow riffles and runs; (2) gear adaptation, dynamically matching collection gears to the stream matrix by deploying a cast net within deeper pool sections and a kick net in shallow, higher-velocity riffles and runs; and (3) effort baseline, maintaining a consistent capture window of more than 2 h per sampling event to secure comparative quantitative coverage across the survey period. The structured allocation of the 676 collected specimens—consisting of 526 individuals captured within deep pool zones and 150 individuals from shallow riffles and runs—along with their respective microhabitat-specific total length and body weight ranges, is comprehensively summarized in Table S3. Captured specimens were identified to the species level on-site; total length (TL) was measured to the nearest 0.01 mm using digital Vernier calipers, and total body weight (BW) was recorded to the nearest 0.1 g using a high-precision electronic balance. Following morphometric processing, the majority of individuals were immediately released back into their respective catchments to minimize mortality and ecological disturbance, while a small subset of specimens was preserved in 80% ethanol for genetic analysis. To resolve any potential taxonomic ambiguities arising from the close morphological proximity among congeneric *Squalidus* species, the identity of this translocated population was independently verified as *S. multimaculatus* through complete mitochondrial genome sequencing (GenBank Accession No. KX49560), as detailed in our associated molecular frameworks [38].

#### 2.4.2. Length–Weight Relationship

We analyzed the length–weight relationship of *S. multimaculatus* as follows:

*W* = *aL^b^*, (1)

where *W* is the body weight (g), *L* is the total length (mm), *a* is the scaling factor, and *b* is the allometric growth coefficient [39]. We estimated the parameters using non-linear regression to avoid potential biases introduced by transformations.

#### 2.4.3. Condition Factor

We evaluated the physiological health and seasonal well-being of the population using Fulton’s condition factor (*K_F_*). We calculated *K_F_* according to the following formula [39]:

*K_F_* = *W*/*L*^3^ × 10^5^, (2)

where *W* is the body weight (g) and *L* is the total length (mm). We analyzed the monthly fluctuations in *K_F_* values to infer the energetic costs and recovery patterns associated with spawning activities and environmental conditions. Specifically, a rapid decline in the condition factor following a distinct peak can reflect the release of gonadal products and the expenditure of somatic energy [40]; therefore, we considered this period to represent an inferred spawning season.

#### 2.4.4. Age Structure

We estimated the age structure of the population using length–frequency distribution (LFD) analysis [41]. To minimize sampling bias and ensure statistical robustness, we reconstructed the monthly length data into fixed frequency tables with 2 mm size intervals ranging from 21 mm to 105 mm, assigning a count of zero to length bins with no observed individuals.

We identified putative age cohorts by performing normal mixture analysis on these fixed LFD tables using the “mixdist” package (version 0.5-5) in R [42]. The number of age components and their initial parameter values (mean *μ*, standard deviation *σ*, and relative proportion *π*) were designated based on the monthly modal shifts observed in the histograms. Where necessary to achieve mathematical convergence and resolve singularity errors caused by rare size classes (e.g., newly recruited 0+ individuals or rare older cohorts), we selectively fixed the standard deviation (*σ*) parameters of specific components during the maximum-likelihood estimation process; therefore, these age assignments should be interpreted as model-based cohort estimates. The specific cohorts with fixed *σ* values, along with their final estimated parameters, are explicitly detailed in the results. We validated the goodness of fit for each monthly mixture model using the Chi-square (*χ*^2^) test, accepting models with a non-significant result (*p* > 0.05).

#### 2.4.5. von Bertalanffy Growth Function

We modeled the growth trajectory of *S. multimaculatus* using the standard von Bertalanffy growth function (VBGF) [43]:

*L_t_* = *L*_∞_ (1 − exp(−*K*(*t* − *t*_0_))), (3)

where *L_t_* is the total length (mm) at age *t* (years), *L*_∞_ is the asymptotic theoretical length, *K* is the growth coefficient, and *t*_0_ is the theoretical age at length zero.

To improve biological realism and stabilize the model at early life stages, we took the initial length at hatching (*L*_0_ = 2.8 mm) based on the morphological development literature of the species [29]. We then rearranged the VBGF as follows to eliminate the parameter *t*_0_ and reduce inter-parameter correlation:

*L_t_* = *L*_∞_ − (*L*_∞_ − 2.8) × exp(*−Kt*), (4)

To assign a fractional age (*t*) to each monthly cohort identified from the mixture analysis, we set 1 July, the midpoint of the inferred June–July period characterized by a rapid decline in the condition factor (*K_F_*), as the age origin (*t* = 0). The time elapsed from this spawning origin to each sampling date was converted into a decimal fraction of a year. To minimize potential bias arising from sample size variations, we calculated the mean length of individuals within the same age class collected on the exact same date and utilized these aggregated mean values to estimate the growth equation. We estimated the final growth parameters (*L*_∞_ and *K*) through non-linear regression using the “nls” function in R.

### 2.5. Statistical Analysis

All statistical analyses and spatial visualizations were performed using the R statistical computing environment (version 4.4.1). The primary aim and purpose of our statistical framework were to evaluate the biogeochemical conditions of the newly colonized Goseong habitat, to identify the core macro-climatic drivers enabling the species’ northward establishment, and to confirm the demographic viability of the translocated population. Specifically, the targets of this analysis encompassed the 29-year continuous environmental monitoring time series (1997–2025) contrasting the historical native benchmarks against the colonized baselines, as well as the biological morphometric dataset obtained from 676 field-sampled individuals.

By executing this targeted framework, we aimed to highlight several key findings mapped to specific statistical models. To highlight discrete group-level environmental and thermal variations across sequential historical phases (Y-*T*_0_, G-*T*_0_, G-*T*_1_, and G-*T*_2_), non-parametric Kruskal–Wallis rank-sum tests followed by Dunn’s post hoc tests with Bonferroni correction were applied. To highlight continuous, long-term warming trajectories and water quality trends without serial autocorrelation bias, formal Seasonal Mann–Kendall and standard Mann–Kendall trend tests accompanied by Sen’s slope estimators were conducted. Furthermore, to highlight the demographic stabilization and somatic condition of the colonized population, age structures were resolved via normal length–frequency mixture modeling, and somatic growth trajectories were modeled using non-linear regressions of the von Bertalanffy growth function (VBGF). Statistical significance was uniformly established at α = 0.05 (*p* < 0.05) across all analyses unless otherwise specified.

## 3. Results

### 3.1. Spatio-Temporal Distribution and Range Expansion

**Figure 2:**
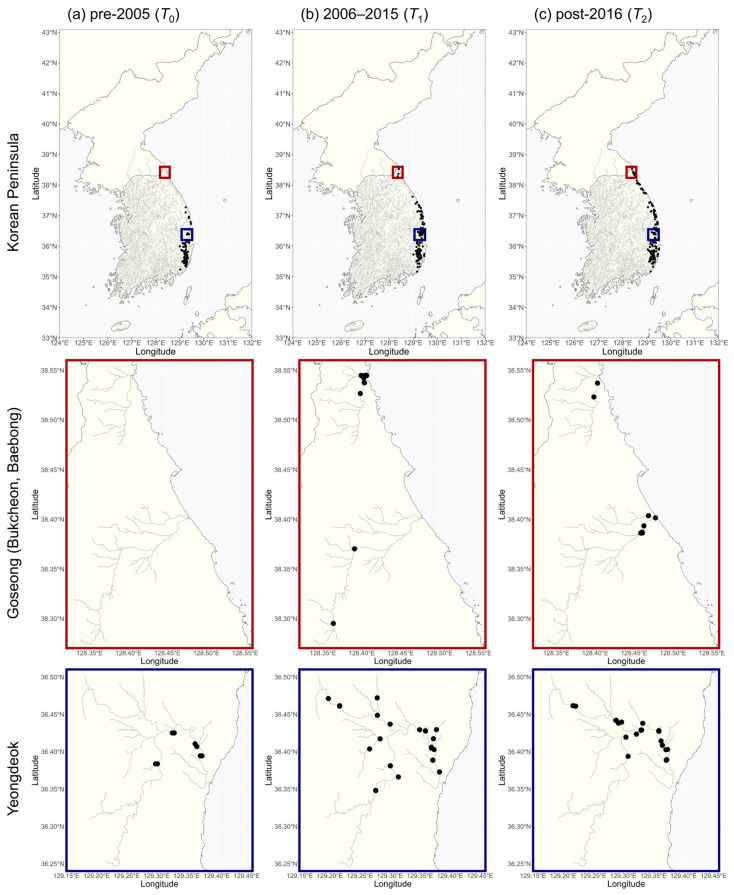
Spatio-temporal shifts in the nationwide distribution of *Squalidus multimaculatus* across the Korean Peninsula over 3 distinct phases: (**a**) pre-colonization (pre-2005, *T*_0_), (**b**) early emergence (2006–2015, *T*_1_), and (**c**) recent establishment (post-2016, *T*_2_). Black dots indicate the recorded occurrence locations. The geographical occurrence dataset was consolidated from the National Ecosystem Survey and Wetland Information System (National Institute of Ecology), the Aquatic Ecosystem Health Assessment (National Institute of Environmental Research), the Biodiversity of the Korean Peninsula database (National Institute of Biological Resources), the Global Biodiversity Information Facility (GBIF), and verified regional ichthyofaunal literature for Goseong catchments [23,24,25,30].

Nationwide occurrence data revealed a progressive northward shift in *S. multimaculatus* along the eastern coast of the Korean Peninsula (Figure 2). Prior to 2005 (*T*_0_), the species’ distribution was restricted to the southeastern coastal drainages. The occurrence records were primarily clustered around Yeongdeok and adjacent southern streams, generally confined to latitudes below 36.5° N. Between 2006 and 2015 (*T*_1_), the occurrence records shifted northward, with new populations appearing at higher latitudes, specifically 38.54° N (our study site; Baebong Stream) and 38.40° N (Bukcheon Stream). Since 2016 (*T*_2_), a continuous distribution of *S. multimaculatus* was recorded across several northern coastal streams, including the Baebong Stream and the Bukcheon Stream in Goseong.

### 3.2. Environmental Conditions and Habitat Suitability

#### 3.2.1. Comparison of Biogeochemical, Physical, and Microbiological Water Quality Baselines

We compared the biogeochemical, physical, and microbiological water quality of the native baseline (Y-*T*_0_) against the 3 spatio-temporal phases of the Goseong habitat (G-*T*_0_, G-*T*_1_, and G-*T*_2_) (Table 1). The Kruskal–Wallis test revealed statistically significant differences across the temporal groups for 6 of the 8 environmental parameters (*p* < 0.05), with the exceptions of SS (*p* = 0.5211) and Total_Coli (*p* = 0.8645). Specifically, Y-*T*_0_ exhibited higher concentrations of TN and TP compared to all Goseong phases, recording 2.99 mg/L (Grade VI) and 0.109 mg/L (Grade II), respectively. Fecal_Coli was also highest in Y-*T*_0_ at 565.11 MPN/100 mL (Grade III) and significantly decreased across the Goseong phases, reaching 35.13 MPN/100 mL (Grade Ia) in G-*T*_2_ (*p* = 0.0005). Based on the statutory water quality classifications, the Goseong habitat phases (G-*T*_0_ to G-*T*_2_) predominantly maintained Grade Ia for parameters including BOD, DO, and TP. At the same time, COD levels were classified as Grade Ia during G-*T*_0_ (1.69 mg/L) and G-*T*_1_ (1.99 mg/L), and Grade Ib in G-*T*_2_ (2.58 mg/L).

We evaluated the 29-year Seasonal Mann–Kendall trends across the 9 environmental variables in Goseong (Table S4). We confirmed a long-term significant increasing trend in Wtemp (*τ* = 0.111, Sen’s slope = 0.0667 °C/year, *p* = 0.0039) and DO (*τ* = 0.138, slope = 0.0250 mg/L/year, *p* = 0.0003). Conversely, we identified a significant decreasing trend in Total_Coli (*τ* = −0.156, slope = −16.8615 MPN/100 mL/year, *p* < 0.0001), while BOD and TP stably maintained statutory Grade Ia thresholds.

#### 3.2.2. Shift in Water Temperature and Critical Cold Stress

The Kruskal–Wallis test indicated statistically significant differences across the temporal groups for all thermal variables (*p* < 0.05), except for Max_Wtemp (*p* = 0.4490) (Table 2). According to Dunn’s post hoc analysis, Mean_Atemp and Min_Atemp in the G-*T*_0_* and G-*T*_1_ phases were significantly lower than Y-*T*_0_*, whereas the G-*T*_2_ phase showed no significant difference from Y-*T*_0_*. For Max_Atemp, a significant difference from Y-*T*_0_* was observed only in the G-*T*_0_* phase, with no significant differences detected in the G-*T*_1_ and G-*T*_2_ phases. Regarding water temperature, Mean_Wtemp in G-*T*_0_ and G-*T*_1_ was significantly lower than in Y-*T*_0_, whereas G-*T*_2_ showed no significant difference (*p* = 0.3733). Min_Wtemp in G-*T*_0_ was significantly lower than Y-*T*_0_, but both G-*T*_1_ and G-*T*_2_ exhibited no significant difference from Y-*T*_0_ (*p* > 0.9999).

We evaluated the 29-year standard Mann–Kendall trends across the aquatic thermal indices in Goseong (Table S4). We confirmed a sharp, significant long-term increasing trend in Min_Wtemp (*τ* = 0.443, Sen’s slope = 0.1191 °C/year, *p* = 0.0007), representing a cumulative baseline rise of ~3.45 °C over the tracking period. Conversely, we observed no significant trend in Max_Wtemp (*τ* = 0.022, slope = 0.0000, *p* = 0.8801).

**Figure 3:**
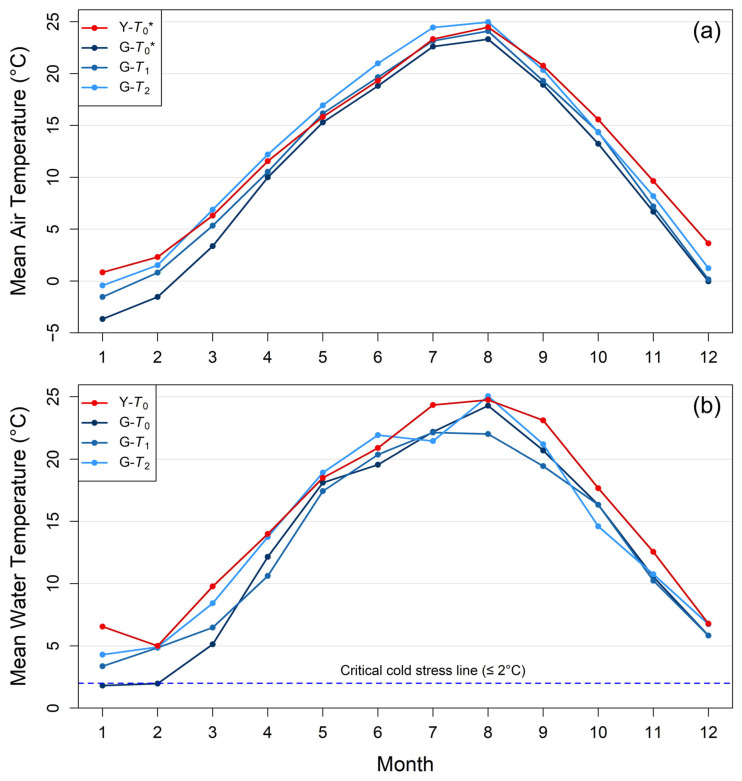
Monthly variations in (**a**) mean air temperature and (**b**) mean water temperature across the native baseline and the temporal phases of the colonized habitat. The blue dashed line in panel (**b**) indicates the critical cold-stress threshold (≤2.0 °C) for the overwintering survival of *Squalidus multimaculatus*. Asterisks (*) in panel (**a**) denote the extended baseline period (1940–2005) for air temperature to capture long-term macroclimatic trends (Y-*T*_0_* and G-*T*_0_*), which differs from the 1997–2005 baseline used for water temperature (Y-*T*_0_ and G-*T*_0_).

The monthly variations in the air and water temperatures are presented in Figure 3. The RMSE for air temperature between the Goseong phases and Y-*T*_0_* decreased over the sequential periods, with values of 2.58 in G-*T*_0_*, 1.63 in G-*T*_1_, and 1.23 in G-*T*_2_. Similarly, the RMSE for water temperature relative to Y-*T*_0_ decreased from 2.52 in G-*T*_0_ to 2.38 in G-*T*_1_, and then to 1.66 in G-*T*_2_. For the cold-stress threshold (water temperature ≤ 2.0 °C), the critical cold-stress ratio in Y-*T*_0_ was 11.1% (Table 3). In Goseong, this ratio was recorded at 40.0% during the G-*T*_0_ phase. In subsequent phases, the critical cold-stress ratio decreased to 3.6% in G-*T*_1_ and 6.9% in G-*T*_2_.

For the cold-stress thresholds, the sensitivity analysis indicated variations across all tested temperatures (Table 3). Extreme cold events (≤1.0 °C) completely disappeared in the colonized habitat, dropping from 28.0% in G-*T*_0_ to 0.0% in both G-*T*_1_ and G-*T*_2_. At the baseline threshold (≤2.0 °C), the ratio was 11.1% in Y-*T*_0_, whereas in Goseong, it recorded 40.0% during G-*T*_0_ before decreasing to 3.6% in G-*T*_1_ and 6.9% in G-*T*_2_. Similarly, the occurrence of events at the ≤3.0 °C threshold precipitously declined from 64.0% in G-*T*_0_ to 13.8% in G-*T*_2_.

### 3.3. Biological Characteristics of the Colonized Population in Goseong

#### 3.3.1. Length–Weight Relationship

**Figure 4:**
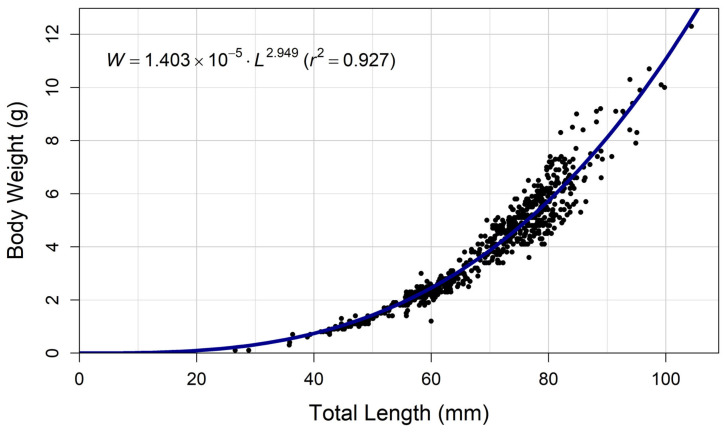
Length–weight relationship of *Squalidus multimaculatus* collected from the colonized habitat (the Baebong Stream in Goseong). Black dots represent individual observations (*n* = 676), and the solid blue curve indicates the fitted non-linear regression model.

A total of 676 *S. multimaculatus* (TL: 26.60–104.39 mm; BW: 0.1–12.3 g) were collected from the Baebong Stream in Goseong. The non-linear regression analysis of the length–weight relationship yielded the following equation (Figure 4):

*W* = 1.403 × 10*^−^*^5^ *L*^2.949^ (*r*^2^ = 0.927, *n* = 676). (5)

The estimated growth coefficient (*b* = 2.949) indicated an isometric growth pattern.

#### 3.3.2. Condition Factor (K_F_)

**Figure 5:**
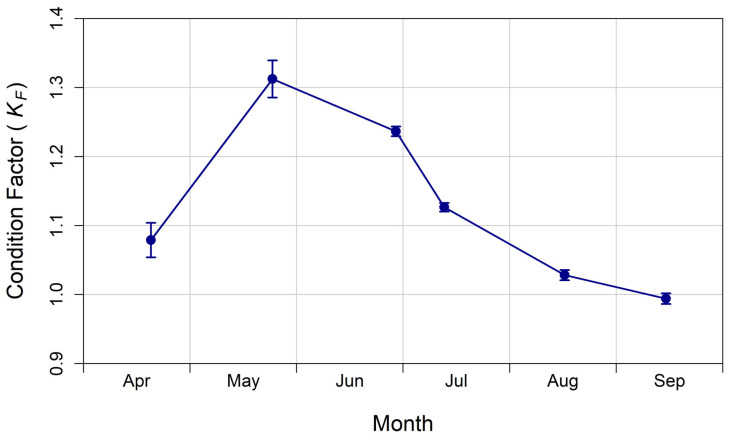
Monthly variations in the condition factor (*K_F_*) of *Squalidus multimaculatus* collected from the colonized habitat (the Baebong Stream in Goseong). Blue dots represent the mean *K_F_* values for each sampling event, and error bars indicate the standard deviation. The horizontal positions of the data points along the *x*-axis are plotted using exact fractional sampling dates to accurately reflect the continuous temporal progression, rather than being centered on discrete monthly categories. Please note that field sampling was exclusively conducted from April to September; therefore, data for the winter months are not available.

The mean condition factor (*K_F_*) for the analyzed *S. multimaculatus* individuals (*n* = 676) was 1.12 ± 0.13 (mean ± SD). The monthly variation in *K_F_* was statistically significant (one-way ANOVA, *F*_5, 670_ = 122.5, *p* < 0.001) (Figure 5). Within the surveyed period from April to September, the mean *K_F_* reached a distinct peak at the end of May (approximately 1.31). Following this peak, a continuous and significant decline was observed throughout the summer months, with the value dropping to approximately 1.03 in August and reaching a minimum of 0.99 in September (Tukey HSD, *p* < 0.05 for all subsequent months vs. May).

#### 3.3.3. Age Structure

**Figure 6:**
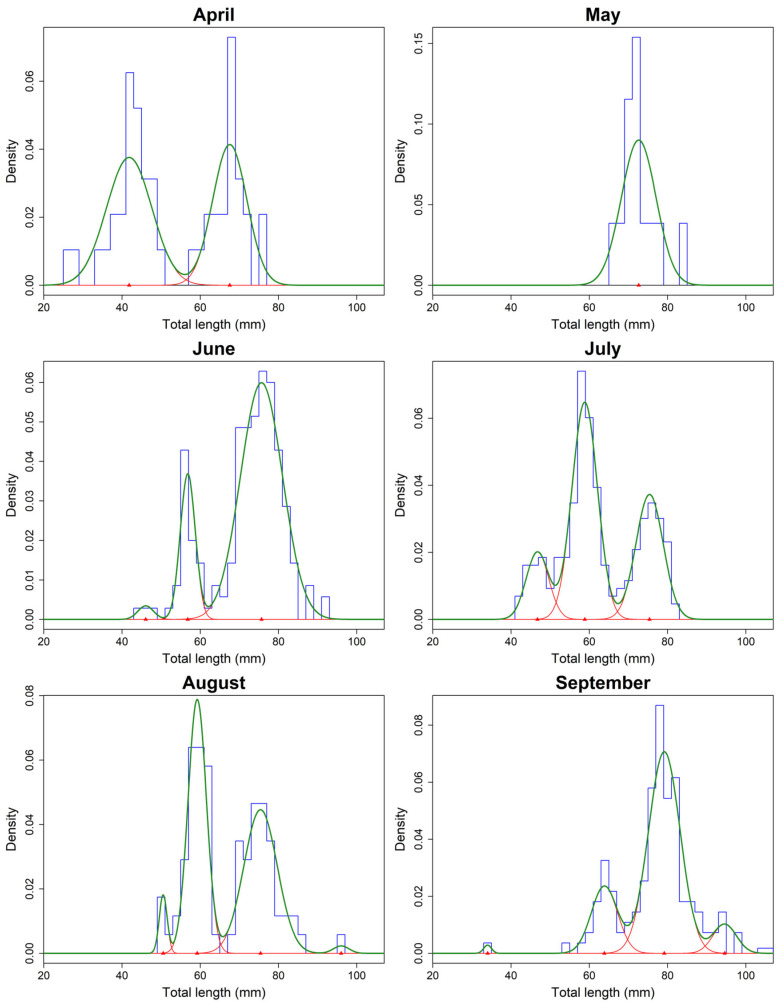
Length–frequency distributions and normal mixture model fits for *Squalidus multimaculatus* across the sampling months (April to September). The blue histograms represent the observed length–frequency density. The green curves indicate the overall fitted normal mixture distributions, and the red curves (with red triangles at the means) represent the estimated individual age cohorts.

The normal mixture analysis conducted for each sampling month resolved model-based putative age cohorts (Age 0+ to 4+) within the *S. multimaculatus* population (Figure 6, Table 4). All monthly models demonstrated acceptable goodness of fit, with Chi-square (*χ*^2^) tests consistently yielding non-significant results (*p* > 0.05). It should be noted that the non-detection of Age 0+ and Age 1+ cohorts in the May sample is attributable to gear selectivity and sampling effort constraints within specific micro-habitats during that month’s survey, rather than a true ecological absence from the population. The cohort structure suggested seasonal shifts in size classes. Notably, older size classes (Age 3+ and 4+) were captured in distinct peaks from July to September, with the Age 4+ group exhibiting mean lengths of 96.00 ± 2.00 mm in August and 94.57 ± 3.00 mm in September. Furthermore, in September, a putative Age 0+ cohort with a mean total length of 34.00 ± 1.00 mm resolved as a distinct component in the length–frequency distribution.

#### 3.3.4. Growth Parameters (VBGF)

**Figure 7:**
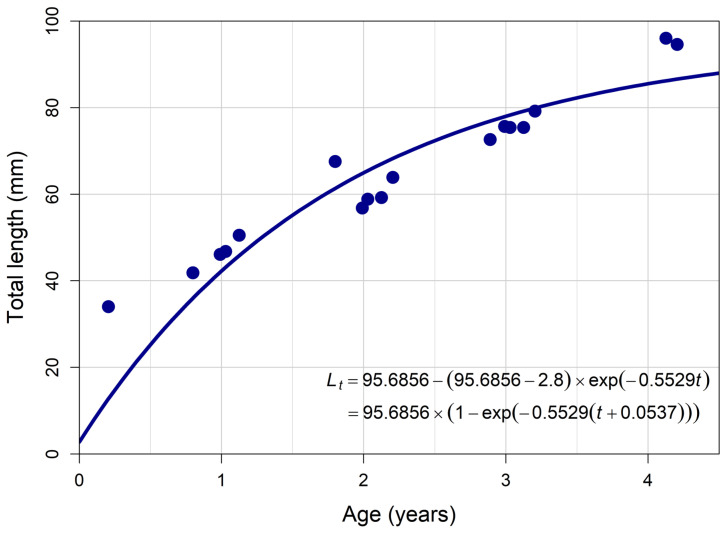
The von Bertalanffy growth curve for *Squalidus multimaculatus* collected from the colonized habitat (the Baebong Stream in Goseong). The blue dots represent the observed mean total lengths for each age cohort across the sampling months. The solid blue curve indicates the estimated theoretical growth trajectory. The specific growth equations, presented in both the standard von Bertalanffy growth function (VBGF) format (Equation (3)) and its exact derived format (Equation (4)), are explicitly displayed within the plot.

The growth of *S. multimaculatus* was evaluated using the von Bertalanffy growth function (VBGF). By fixing the initial length at hatching (*L*_0_) at 2.8 mm, the non-linear regression estimated the asymptotic length (*L*_∞_) to be 95.69 ± 9.18 mm, and the growth coefficient (*K*) was 0.55 ± 0.12 year^−1^ (*p* < 0.001). The residual standard error of the model was recorded as 8.04. The estimated growth curve and the observed mean lengths of each putative age cohort across the sampling months are presented in Figure 7.

## 4. Discussion

### 4.1. Post-Translocation Establishment and Environmental Suitability of Squalidus multimaculatus

The spatial analysis confirms that the geographical range of *S. multimaculatus* has progressively expanded from streams draining into the southeastern coast to those flowing into the northeastern coast of the Korean Peninsula (Figure 2). Notably, the new population identified in Goseong following the initial temporal phase is established within an independent drainage network completely isolated from the historical native catchments (Figure 2). Although nationwide ichthyofaunal surveys conducted by the Ministry of Environment have been continuously carried out in these northernmost streams since 1987 (https://www.nie-ecobank.kr, accessed on 30 May 2026), *S. multimaculatus* was not detected in Goseong until 2008 (Figure 2). Since 2009, however, the species has been consistently reported in the region (Figure 2), a phenomenon primarily attributed to anthropogenic translocation and stocking aimed at recovering the fish resource following severe typhoon-induced habitat disruption [24,26,30]. An assessment of local fish assemblages in 2013 classified *S. multimaculatus* as a subdominant species in the lower reaches of the Baebong Stream in Goseong, providing early evidence of its successful colonization and population stabilization [24]. Furthermore, our recent comprehensive dataset demonstrates that the species is currently documented across numerous independent coastal streams spanning the entire eastern longitudinal gradient of the Republic of Korea, indicating a notable shift in its biogeographical distribution (Figure 2). Importantly, our analytical framework remains highly robust. The expanded dataset, in conjunction with our empirical fieldwork, not only confirms the continuous, multi-generational survival of the species within the Baebong Stream from *T*_1_ through *T*_2_ but also supports its established occurrence in the Bukcheon Stream during *T*_2_, thereby validating the use of the adjacent Bukcheon Stream as a valid environmental proxy.

Although the growth and distribution of freshwater fish are governed by a complex array of physicochemical factors, this study focused on evaluating long-term biogeochemical trends, leaving precipitation-driven fluctuations in flow velocity outside the immediate scope of this analysis. Nevertheless, the fundamental physical template of the habitat was explicitly verified through geomorphological baselines (Table S1). The lower reaches of the Goseong catchments feature similar Bc-type river morphologies characterized by heterogeneous mixed substrates—such as the pebble-dominant matrix in the Baebong Stream—which functionally accommodate the benthic-sifting and ontogenetic sheltering requirements of *S. multimaculatus* (Table S3). In tandem with this physical comparability, the core water quality indices—including BOD, COD, DO, SS, TN, and TP—in the newly colonized Goseong habitat were generally maintained at levels lower than or comparable to those recorded in the historical native habitat of Yeongdeok. Among these variables, dissolved oxygen (DO) is a key constraint for freshwater fishes, directly governing metabolic scope, respiratory efficiency, and survival, particularly during peak summer metabolic demands [44]. In freshwater river networks, total phosphorus (TP) is widely recognized as the primary limiting nutrient triggering cultural eutrophication [45]. In Goseong, maintaining high and stable DO concentrations is fundamentally safeguarded by consistently low TP loading, which suppresses excessive algal proliferation and subsequent microbial oxygen depletion. This persistent baseline stability indicates that Goseong provides generally favorable biogeochemical conditions, supporting the multi-generational establishment and long-term persistence of *S. multimaculatus* despite unmeasured physical variations in river hydrodynamics.

The northward range expansion of freshwater fish can serve as indicators of climate-related environmental change, often associated with the structural relaxation of thermal constraints in previously uninhabitable high-latitude regions [3,46]. The critical thermal minimum for various temperate cyprinid species has been reported to range approximately from 2.0 °C to 6.0 °C, varying dynamically with their thermal acclimation history [37]. This empirical baseline suggests that the historical winter water temperatures during the pre-colonization phase in Goseong (G-*T*_0_) frequently fell below the estimated physiological tolerance threshold of *S. multimaculatus*. Such recurrent exposure to sub-threshold temperatures would have imposed severe, chronic cold stress, thereby acting as a critical thermal barrier that historically restricted the species’ northward range establishment [3,18,37]. Although the exact physiological limit of *S. multimaculatus* remains unexplored, our sensitivity analysis demonstrates that the macro-climatic trend is robust regardless of the specific thermal threshold.

During the recent establishment period (G-*T*_2_), the occurrence ratio of critical cold stress (≤2.0 °C) during the winter season declined from 40.0% to 6.9% (Table 3). Although a slight non-monotonic fluctuation was observed at this threshold between G-*T*_1_ (3.6%) to G-*T*_2_ (6.9%), this minor rebound falls well within natural inter-annual climate variability. Both recent phases represent a fundamental structural alleviation of cold stress compared to the historical baseline. This overarching warming trajectory is further corroborated by our sensitivity analysis, which revealed a strictly monotonic decrease at the ≤3.0 °C threshold (precipitously declining from 64.0% in G-*T*_0_ to 13.8% in G-*T*_2_) and the complete disappearance of extreme cold events (≤1.0 °C). Notably, our long-term Mann–Kendall trend analysis supports the possibility of this mechanistic premise, suggesting that the localized thermal regime shift may be primarily driven by the lifting of winter cold extremes (Min_Wtemp: Sen’s slope = 0.1191 °C/year, *p* = 0.0007) rather than summer heating (Max_Wtemp: *p* = 0.8801). This macro-climatic transition is quantitatively supported by the convergence of aquatic and atmospheric thermal profiles over the sequential periods. The root-mean-square error (RMSE) for air temperature between the Goseong phases and the native Yeongdeok baseline (Y-*T*_0_*) decreased from 2.58 in G-*T*_0_* to 1.23 in G-*T*_2_ (Table S5). Similarly, the RMSE for water temperature relative to Y-*T*_0_ contracted from 2.52 to 1.66 (Table S5). Furthermore, recent thermal conditions in G-*T*_2_ exhibited no significant differences from the native baseline across key metrics, including minimum and mean water temperatures (Table 2). This synchronization of the thermal niche suggests that the northeastern coast of the Republic of Korea provides a climatic refugium matching the historical baselines of the species’ native range. While anthropogenic pathways initiated the translocation, regional climate-driven thermal homogenization appears to be the primary mechanism enabling its multi-generational persistence.

**Figure 8:**
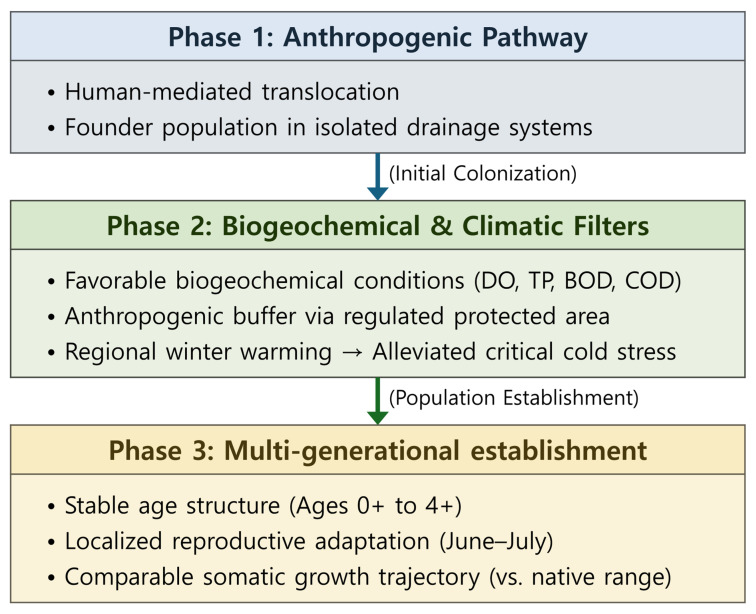
Conceptual framework illustrating the sequential filtering process—comprising the anthropogenic pathway (Phase 1), biogeochemical & climatic filters (Phase 2), and multi-generational establishment (Phase 3)—governing the post-translocation colonization dynamics of *Squalidus multimaculatus*.

This multi-phase biogeochemical and demographic transition is systematically synthesized into a comprehensive conceptual framework (Figure 8). The framework illustrates that the post-translocation process is governed by a series of sequential filtering phases that dictate long-term colonization dynamics. Specifically, Phase 1 (Anthropogenic Pathway) highlights the initial human-mediated introduction that establishes a founder population within hydrologically isolated drainage systems. Phase 2 (Biogeochemical & Climatic Filters) demonstrates how the population navigates localized environmental constraints; this is driven by favorable biogeochemical conditions—predominantly characterized by the multi-decadal stability of DO, TP, BOD, and COD—safeguarded by an anthropogenic buffer via a regulated protected area, combined with regional winter warming trends that functionally alleviate critical winter cold stress. Consequently, Phase 3 (Multi-generational Establishment) suggests the long-term persistence of the species, evidenced by a stable multi-generational age structure (Ages 0+ to 4+), localized reproductive adaptation (June–July), and both a somatic growth performance and a growth trajectory that are comparable to those reported in its native range. The empirical parameters and statistical models validating each conceptual phase are mapped to the corresponding analytical modules established throughout this study (Table 1, Table 2, Table 3 and Table 4 and Table S3, and Figure 2, Figure 3, Figure 5 and Figure 7).

### 4.2. Comparative Population Dynamics and Biological Adaptation of Squalidus multimaculatus

The ultimate evidence of a successful range expansion is the establishment of a self-sustaining reproductive cycle in the new environment [47,48]. Our biological evaluations suggest that the *S. multimaculatus* population in Goseong has adapted to its environment. It is important to note that our empirical biological dataset, collected in 2014, represents a critical mechanistic snapshot of the late stage of the early emergence phase (*T*_1_) rather than the continuous inter-annual dynamics of the recent establishment phase (*T*_2_). Specifically, the presence of an Age 4+ cohort in our 2014 dataset biologically suggests that these individuals had successfully overwintered and reproduced in this novel habitat since at least 2010, thereby providing fundamental evidence of successful early-stage biological assimilation. The ongoing persistence and spatial stability of this multi-generational population throughout the subsequent *T*_2_ phase are independently corroborated by our continuous multi-decadal occurrence tracking (Figure 2). The mean condition factor (*K_F_*) reached its annual peak in May (1.31 ± 0.10), representing the period of maximum energy accumulation and gonad maturation prior to spawning (Figure 5). This physiological peak was empirically corroborated by our field survey on 24 May, during which we directly observed gravid females in an impending spawning condition. Following this peak, a continuous and significant decline in *K_F_* was observed from June to September (Tukey HSD, *p* < 0.05 for all subsequent months vs. May) (Figure 5). Notably, the most substantial reductions occurred between June and July (mean difference = −0.110, *p* < 0.001). Based on these combined physical observations and physiological metrics, the spawning period for the Goseong population is estimated to occur from June to July. This timeframe aligns with the reproductive phenology of other congeneric species within the genus *Squalidus* (e.g., *S. j. coreanus* and *S. c. tsuchigae*), which are reported to spawn during June and July [49,50]. Interestingly, our estimated spawning season is slightly earlier than that of a native *S. multimaculatus* population in a different region, which was previously reported to spawn from July to August [28]. While not a definitive finding, this observed phenological shift presents a compelling hypothesis for future testing, suggesting that the newly colonized northern population may exhibit localized adaptation in its reproductive timing in response to the local thermal regime. By September, the condition factor reached its lowest value (0.99 ± 0.09), indicating the termination of the spawning period and the onset of a post-spawning recovery phase. This localized reproductive cycle is supported by our age structure analysis, which shows that a newly recruited generation (Age 0+) abruptly emerged in September, confirming successful local recruitment (Figure 6).

Beyond reproductive phenology, the length–weight relationship (LWR) and the baseline condition factor (*K_F_*) provide supporting evidence for the somatic condition of the population in the newly colonized habitat. The LWR analysis yielded a high coefficient of determination (*r*^2^ = 0.927), with an estimated growth parameter (*b*) of 2.949 (Figure 4). In fish population dynamics, the *b* parameter serves as a critical indicator of growth patterns and environmental suitability [39]. Jo et al. (2018) reported a *b* value of 3.18 for this species, but this prior estimate was derived from a highly limited sample size (*n* = 6) and a narrow length range [51]. In contrast, our extensive dataset (*n* = 676), encompassing a comprehensive ontogenetic length range from 26.60 to 104.39 mm, provides a highly robust and representative population-level estimate (Figure 4). The observed *b* value reflects an isometric growth pattern, suggesting proportional somatic growth. Furthermore, the generally robust overall condition factor (mean *K_F_* = 1.12 ± 0.14) maintained across the population indicates that the streams offer abundant prey resources and highly favorable foraging conditions [39,40]. These metrics suggest that the individuals were not experiencing severe somatic constraints. This high energetic efficiency directly provides the physiological foundation for the robust long-term growth trajectories of the population.

This study provides the first formal estimation of the von Bertalanffy growth parameters for *S. multimaculatus* (Figure 7). The previous literature on native populations relied primarily on the Petersen method (length–frequency distribution) to estimate age, reporting empirical length intervals rather than fitting a continuous non-linear growth model [28]. According to these prior records, the native population was characterized by length classes of <50 mm for 1-year-olds, 50–69 mm for 2-year-olds, 70–89 mm for 3-year-olds, and >90 mm for 4-year-olds, with a maximum observed length of approximately 95–100 mm TL [28]. Our VBGF estimates for the newly colonized Goseong population are consistent with these previously documented baselines (Figure 7). Specifically, the theoretical lengths calculated from our model are 42.25 mm at 1 year, 64.95 mm at 2 years, and 78.00 mm at 3 years, all of which fall well within the reported native length-at-age boundaries. Furthermore, the calculated length of 85.51 mm at 4 years smoothly transitions toward the previously documented >90 mm threshold, ultimately converging with the theoretical asymptotic length (*L*_∞_ = 95.69 ± 9.18 mm TL), which coincides with the maximum limit reported for native populations. This theoretical limit is consistent with our age structure analysis, which successfully captured an Age 4+ cohort reaching 94.57–96.00 mm in late summer (Table 4). Furthermore, the mean lengths of our younger age cohorts align with the established growth benchmarks of the native range (Figure 7). This structural and dimensional congruence in growth suggests that the northern streams provide favorable nutritional and thermal conditions, allowing the colonized population to achieve successful biological establishment and exhibit growth vitality comparable to its native benchmarks [18,52].

Furthermore, our biological evaluations of the early life stages exhibit remarkable congruence with the established ontogenetic trajectories of the species. A previous laboratory rearing study reported that newly hatched *S. multimaculatus* larvae have a mean total length of 2.8 mm [29], a physiological baseline that we successfully incorporated as the theoretical length at hatching (*L*_0_) in our VBGF model. More importantly, the controlled experiment demonstrated that juveniles reach a mean length of 32.1 mm at 80 days post-hatching [29]. This empirical timeline is broadly consistent with our field observations. Calculated from our methodologically designated age origin (1 July) and the exact field sampling date (14 September), the newly recruited Age 0+ cohort (mean length 34.00 ± 1.00 mm) approximately corresponds to individuals around 75 days post-hatching under our assumed age-origin framework (Table 4). The fact that the physical size of our 75-day-old wild cohort closely mirrors the 80-day-old laboratory-reared juveniles serves a dual purpose. It supports the plausibility of local early-life development in Goseong habitat and provides indirect support for using 1 July as the age origin.

### 4.3. Ecological Implications and Future Perspectives on Predictive Modeling

A primary objective of conservation biology is to decipher how species have historically responded to climate-induced environmental transitions to accurately anticipate their future distributional trajectories [53]. However, contemporary predictive modeling exhibits a disproportionate reliance on forward-looking simulations (forecasting) while largely neglecting the multi-decadal empirical responses that have already unfolded (empirical baseline) [14]. This methodological omission risks ignoring the immediate footprints of rapid ongoing climate change, thereby escalating predictive uncertainty in future habitat assessments [54,55]. Addressing this critical knowledge gap, this study successfully elucidated the long-term, quantitative mechanisms governing the air-water-biota continuum during the range reconfiguration of *S. multimaculatus*. The high-resolution empirical baselines established herein serve as an invaluable asset for refining climate-scenario-based predictive frameworks, effectively bridging historical demographic shifts with future habitat suitability projections.

The climate-driven range dynamics of *S. multimaculatus* demonstrated in this study align precisely with core thermal ecology frameworks, which posit that species possessing higher optimal growth temperatures can capitalize on warming regimes as ecological opportunities for range expansion [46,55]. Within the context of the thermal performance curve, *S. multimaculatus* is recognized as one of the endemic freshwater fishes with the highest optimal growth thresholds in the region, offering it a distinct metabolic advantage under escalating global warming scenarios [52]. Critically, the historical winter thermal barrier—which once acted as a definitive physiological bottleneck precluding overwintering survival—has structurally collapsed through a step-wise regime shift, even at the Republic of Korea’s highest latitude. Consequently, from a purely thermal standpoint, the entire eastern corridor of the peninsula has shifted into a viable climate refuge.

Nevertheless, the geographical expansion of climatically suitable niches does not inherently guarantee long-term population persistence. Endemic species tightly bound to downstream reaches of river networks remain highly vulnerable to localized, human-induced environmental degradation within their newly acquired frontiers [56]. Beyond macro-climatic drivers, fragmented river connectivity caused by dams and weirs, sudden water quality deterioration, and the aggressive invasion of exotic species act as compounding anthropogenic stressors that can accelerate the localized extirpation of native populations [13,57,58]. Therefore, the effective conservation of indigenous freshwater fish demands a proactive management paradigm that prioritizes the remediation of artificial migration barriers and the restoration of degraded instream habitats. Because the extirpation of an endemic species represents an irreversible global extinction rather than a localized loss, establishing a scientifically robust, data-driven protection framework rooted in long-term habitat and population analysis is of paramount importance [57,59]. Ultimately, integrating our empirical ecological-environmental framework will facilitate the strategic implementation of advanced conservation tools, such as managed translocation and assisted migration, to safeguard endangered endemic populations against accelerating global change [60].

Finally, while this study successfully prioritizes the eco-physiological adjustments of a single endemic species, climatic range shifts in freshwater ecosystems often manifest with high interspecific variations and complex community-level reconfigurations [3,53]. For instance, multi-decadal climate tracking in temperate Ontario lakes revealed that different trophic guilds respond asynchronously to warming velocities, where expanding apex predators shift northward, whereas lower-trophic prey fishes frequently experience localized extirpations [61]. Given that *S. multimaculatus* has achieved successful demographic integration in the lower reaches of Goseong [24], future long-term monitoring must expand beyond single-species demographic traits. Integrating community-level trophic webs and evaluating potential competitive shifts between expanded endemics and indigenous residents will be imperative to fully understand the systemic ecological consequences of post-translocation range alterations. In this regard, the integrated air–water–biota dynamics summarized within this framework (Figure 8) serve as a foundational benchmark to guide and refine such future predictive and management strategies.

### 4.4. Methodological Limitations and Future Recommendations for Post-Translocation Research

While our empirical baseline framework successfully links climatic biogeochemistry and warming with the post-translocation establishment of *S. multimaculatus*, several methodological limitations remain. First, our biological dataset represents a single-year snapshot (2014), restricting direct inferences on multi-decadal population dynamics. To mitigate this constraint, our quality-controlled mapping integrating multi-agency databases and regional literature (Figure 2) independent of the 2014 fieldwork explicitly validates continuous, stable occurrence in both Baebong and Bukcheon Streams throughout the recent *T*_2_ phase (post-2016), confirming sustained persistence rather than a transient emergence despite unquantified historical survey effort variations.

Second, demographic and phenological traits were inferred through indirect diagnostics. The length–frequency age structures are model-dependent due to selectively fixed standard deviations and require future validation via calcified structures like otoliths [41,62,63]. Similarly, the June–July spawning window inferred from condition factor shifts remains provisional and requires validation using direct reproductive evidence, particularly gonad histology and targeted larval drift or larval occurrence surveys [64,65]. Additionally, although our multi-tier thermal sensitivity analysis (≤1.0 °C, ≤2.0 °C, and ≤3.0 °C) suggests that the winter warming trend is independent of minor physiological threshold variations (Table 3), monthly aggregates can mask transient, sub-threshold daily minima, necessitating high-resolution in situ loggers in future microclimatic models. Additionally, the temporal asymmetry between the long-term atmospheric baseline (1940–2005) and the shorter aquatic baseline (1997–2005) reflects a deliberate methodological choice to comprehensively capture the historical thermal envelope of *S. multimaculatus* within its long-established native range in Yeongdeok, despite subsequent constraints imposed by national hydrological monitoring infrastructure.

Third, establishing an introduced population fundamentally requires navigating local biotic filters. Drawing on the community data in the Baebong Stream from Ko et al. (2013), *S. multimaculatus* exhibits substantial spatial and dietary niche overlap with native benthic residents (*Rhynchocypris steindachneri* and *Misgurnus anguillicaudatus*) within the Bc-type reach matrix [24]. Rather than a simple product of shifting distribution boundaries, actual post-translocation persistence is likely influenced by a complex interplay between abiotic micro-refugia—such as the velocity-mitigated muddy pools and structurally complex pebbly riffles that accommodate ontogenetic sheltering requirements (Table S3)—and biotic resistance from indigenous resident assemblages [47,48]. To refine this predictive framework under accelerating global change, future research must shift from single-species profiling toward: (1) high-density genome-wide SNPs or population genomics to precisely trace fine-scale micro-evolutionary source pathways and localized founder effects within this newly established frontier, expanding upon the existing microsatellite frameworks [22,26]; and (2) community-level trophic web quantification to assess actual resource-partitioning dynamics and systemic ecological integration risks.

## 5. Conclusions

This study provides an empirical baseline linking long-term macroclimatic changes and regional biogeochemical suitability with the multi-generational establishment of the endemic cyprinid *Squalidus multimaculatus* at its northernmost range limit. Our environmental and thermal assessments indicate that while the Goseong coastal stream ecosystem (monitored via the adjacent Bukcheon Stream proxy) has generally favorable biogeochemical conditions, including high dissolved oxygen concentrations and low nutrient loading (Table 1), historical winter temperatures may have imposed potential cold-stress constraints on population persistence (Table 2; Figure 3). However, regional climate warming has progressively alleviated this thermal limitation, reducing critical cold-stress events (≤2.0 °C) from 40.0% to 6.9% and unlocking the northern thermal window decades after initial anthropogenic introduction (Table 3). The biological and demographical analyses of 676 specimens confirm that this thermal niche synchronization facilitated successful biological establishment, as evidenced by a stable age structure (Ages 0+ to 4+), successful local recruitment, a synchronized spawning phenology (June–July), and a robust growth trajectory (*L*_∞_ = 95.69 mm) broadly comparable to native baselines (Figure 5, Figure 6 and Figure 7). Ultimately, this research underscores the importance of incorporating multi-decadal empirical responses rather than relying solely on forward-looking predictive simulations to mitigate uncertainty in species distribution modeling. Although climatic warming has expanded the thermally viable niches for this endemic fish, downstream reaches of independent river networks remain highly vulnerable to compounding localized anthropogenic stressors, including fragmented connectivity, sudden pollution, and invasive species. Therefore, proactive conservation management should consider the remediation of artificial migration barriers and the restoration of degraded instream habitats. The integrative data-driven framework established herein offers an empirical baseline to refine predictive modeling and strategically guide conservation tools, such as managed translocation and assisted migration, to safeguard vulnerable endemic freshwater ichthyofauna in an era of accelerating global change.

## Figures and Tables

**Table 1:** Comparison of biogeochemical, physical, and microbiological water quality parameters and statutory environmental grades between the native baseline (Y-*T*_0_) and the 3 spatio-temporal phases of the colonized habitat (G-*T*_0_, G-*T*_1_, and G-*T*_2_) for *Squalidus multimaculatus*. Values are presented as mean ± standard deviation. The *p*-values indicate the statistical significance across the 4 temporal groups based on the Kruskal–Wallis rank-sum test. Values with different superscript letters (a, b, c) within the same row are significantly different (*p* < 0.05, Dunn’s post hoc test). The environmental grades (Grades Ia to VI) were classified according to the Republic of Korea’s statutory water quality standards. (SS: suspended solids, DO: dissolved oxygen, BOD: biochemical oxygen demand, COD: chemical oxygen demand, TN: total nitrogen, TP: total phosphorus, Fecal_Coli: fecal coliforms, Total_Coli: total coliforms).

Variable	Y-*T*_0_		G-*T*_0_		G-*T*_1_		G-*T*_2_		*p*-Value
	Mean ± SD	Grade	Mean ± SD	Grade	Mean ± SD	Grade	Mean ± SD	Grade	
SS	3.16 ± 1.66	Ia–III	3.95 ± 4.76	Ia–III	4.14 ± 4.86	Ia–III	2.56 ± 0.92	Ia–III	0.5211
DO	11.23 ± 0.48 ^a^	Ia	10.30 ± 0.38 ^b^	Ia	10.85 ± 0.69 ^ab^	Ia	10.74 ± 0.30 ^ab^	Ia	0.0061
BOD	1.20 ± 0.51 ^a^	Ib	0.69 ± 0.05 ^bc^	Ia	0.65 ± 0.16 ^c^	Ia	0.96 ± 0.30 ^ab^	Ia	0.0008
COD *	2.40 ± 0.93 ^ab^	Ib	1.69 ± 0.22 ^a^	Ia	1.99 ± 0.59 ^a^	Ia	2.58 ± 0.27 ^b^	Ib	0.0023
TN **	2.99 ± 0.58 ^a^	VI	1.00 ± 0.13 ^b^	IV	1.32 ± 0.18 ^ab^	V	1.04 ± 0.22 ^b^	V	<0.0001
TP	0.109 ± 0.067 ^a^	II	0.028 ± 0.029 ^b^	Ib	0.014 ± 0.006 ^b^	Ia	0.020 ± 0.004 ^ab^	Ia	0.0008
Fecal_Coli	565.11 ± 508.79 ^a^	III	84.12 ± 117.34 ^b^	Ib	39.03 ± 71.34 ^b^	Ia	35.13 ± 29.92 ^b^	Ia	0.0005
Total_Coli	2176.6 ± 2556.7	III	2678.6 ± 2790.2	III	2784.1 ± 3018.8	III	1780.0 ± 1737.8	III	0.8645

* The environmental grades for COD were determined based on the historical river water quality standards of the Korean Ministry of Environment (prior to the mandatory transition to TOC), which was evaluated to maintain consistency with the multi-decade historical baseline data (Y-*T*_0_). ** Because the river water quality standard in the Republic of Korea does not explicitly designate regulatory thresholds for TN, the TN environmental grades were evaluated based on the National Environmental Standards for Lakes and Reservoirs (Ministry of Environment, Korea) to provide a comparative scale for nutrient loading.

**Table 2:** Spatio-temporal variations in annual air temperature (Atemp) and water temperature (Wtemp) metrics between the native baseline (Y-*T*_0_) and the 3 phases of the colonized habitat (G-*T*_0_, G-*T*_1_, and G-*T*_2_) associated with the distribution of *Squalidus multimaculatus*. Values are presented as mean ± standard deviation. The *p*-values indicate statistical significance across the 4 temporal groups, as determined by the Kruskal–Wallis test. Values with different superscript letters (a, b, c) within the same row are significantly different (*p* < 0.05, Dunn’s post hoc test). (Mean: annual mean temperature, Min: average of the minimum monthly temperatures per year, Max: average of the maximum monthly temperatures per year).

Variable	Y-*T*_0_	G-*T*_0_	G-*T*_1_	G-*T*_2_	*p*-Value
Mean_Atemp	12.80 ± 0.71 ^a^	10.58 ± 0.76 ^c^	11.6 ± 0.47 ^bc^	12.63 ± 0.43 ^ab^	<0.0001
Min_Atemp	0.54 ± 1.71 ^a^	−3.88 ± 2.01 ^c^	−1.80 ± 1.73 ^bc^	−0.75 ± 1.00 ^ab^	<0.0001
Max_Atemp	24.61 ± 1.08 ^a^	23.59 ± 1.17 ^b^	24.47 ± 0.90 ^ab^	25.28 ± 0.88 ^a^	<0.0001
Mean_Wtemp	15.45 ± 1.18 ^a^	13.36 ± 0.82 ^b^	13.38 ± 0.90 ^b^	14.4 ± 1.13 ^ab^	0.0008
Min_Wtemp	3.67 ± 2.12 ^a^	1.39 ± 0.99 ^b^	3.17 ± 0.81 ^a^	3.56 ± 1.51 ^a^	0.0061
Max_Wtemp	25.67 ± 2.74 ^a^	25.61 ± 2.26 ^a^	24.18 ± 2.46 ^a^	25.41 ± 2.36 ^a^	0.4490

**Table 3:** Occurrence ratio (%) of critical cold-stress events (water temperature ≤ 2.0 °C) during the winter season (December to February) across the native baseline (Y-*T*_0_) and the three temporal phases of the colonized habitat (G-*T*_0_, G-*T*_1_, and G-*T*_2_), incorporating a ±1.0 °C sensitivity analysis.

Threshold	Y-*T*_0_	G-*T*_0_	G-*T*_1_	G-*T*_2_
≤1.0 °C	3.7	28.0	0.0	0.0
≤2.0 °C	11.1	40.0	3.6	6.9
≤3.0 °C	29.6	64.0	21.4	13.8

**Table 4:** Monthly estimated mean total lengths (*μ*, mm) and standard deviations (*σ*), and relative proportions (*π*, %) for the identified age cohorts (Age 0+ to Age 4+) of *Squalidus multimaculatus* collected from April to September. The total monthly sample size is denoted by *n*. Cohort values are presented as *μ* ± *σ* (*π*). Standard deviation (*σ*) parameters that were selectively fixed during the mixture model estimation process to achieve mathematical convergence are denoted with an asterisk (*). Hyphens (-) indicate that the corresponding age cohort was not resolved by the monthly mixture model, rather than confirming true absence in the given month.

Month	Age 0+	Age 1+	Age 2+	Age 3+	Age 4+
April (*n* = 48)	41.82 ± 5.79 (54.6%)	67.54 ± 4.38 (45.4%)	-	-	-
May (*n* = 13)	-	-	72.62 ± 4.43 (100%)	-	-
June (*n* = 175)	46.06 ± 2.00 * (1.7%)	56.79 ± 1.91 (17.6%)	75.65 ± 5.37 (80.7%)	-	-
July (*n* = 216)	-	46.74 ± 2.88 (14.6%)	58.83 ± 3.25 (52.8%)	75.38 ± 3.49 (32.6%)	-
August (*n* = 86)	-	50.51 ± 1.00 * (4.6%)	59.21 ± 2.35 (46.4%)	75.40 ± 4.29 (47.9%)	96.00 ± 2.00 * (1.2%)
September (*n* = 138)	34.00 ± 1.00 * (0.7%)	-	63.85 ± 3.34 (19.8%)	79.15 ± 4.05 (71.8%)	94.57 ± 3.00 * (7.7%)

## Data Availability

Data are contained within the article and Supplementary Materials.

## Supplementary Materials

The following supporting information can be downloaded at https://www.mdpi.com/article/10.3390/biology15141140/s1. Table S1: Physiographic, geomorphological, and administrative parameters of Baebong Stream and Bukcheon Stream evaluated at their respective river mouths to justify the spatial proxy framework. Table S2: Statutory water quality standards and parameter thresholds for rivers and streams (living environment) stipulated by the Framework Act on Environmental Policy of the Republic of Korea. Table S3: Quantitative physical metrics, microhabitat dimensions, standardized sampling gear specifications, and summary of the field-derived morphometric dataset for *Squalidus multimaculatus* in Baebong Stream, Goseong. Table S4: Long-term monotonic trend analysis of monthly water quality parameters and annual aquatic thermal indices in the newly colonized Goseong coastal stream habitat. Table S5: Root-mean-square error (RMSE) values for monthly mean air and water temperatures, comparing the native baseline to the three temporal phases of the colonized habitat. Figure S1: Comprehensive cross-validation of daily mean air temperatures across local ground observation stations and the ERA5 reanalysis dataset (2016–2025).
