# Supplementary material for: Post-Translocation Establishment of the Endemic Cyprinid Squalidus multimaculatus Under Favorable Biogeochemical Conditions and Regional Winter Warming
Source: Biology (Basel). 2026 Jul 13;15(14):1140. doi: 10.3390/biology15141140 (PMC13403783; doi:10.3390/biology15141140)
Supplement: Supplementary file 1 [file biology-15-01140-s001.zip › biology-4381547-supplementary.pdf]

Supplementary Material

Table S1. Physiographic, geomorphological, and administrative parameters of Baebong Stream and Bukcheon Stream evaluated at their respective river mouths to justify the spatial proxy framework. The comparative matrix establishes the macroclimatic, micro-habitat, and regulatory congruences between the biological investigation site and the environmental baseline proxy site. (ERA5: European Centre for Medium-Range Weather Forecasts Reanalysis v5, Bc-type: lowermost plain/estuary reach type based on Kani's geomorphological stream classification)

Parameter/feature	Baebong Stream	Bukcheon Stream
Geographic location (at river mouth)	Goseong-gun, Gangwon-do (38.5422°N, 128.4024°E)	Goseong-gun, Gangwon-do (38.4018°N, 128.4775°E)
Macroclimatic grid (at river mouth)	Identical single 0.25° ERA5 Grid	Identical single 0.25° ERA5 Grid
Connected water treatment plant	Hyeonnae-myeon water treatment facility	Goseong Integrated water treatment plant
Water supply protection zone	Designated Hyeonnae Water Supply Protection Area (since 2002)	Designated Ganseong Water Supply Protection Area (since 1987)
Analyzed reach	Lower reaches / downstream	Lower reaches / downstream
River reach type	Bc-type (Lowermost plain type)	Bc-type (Lowermost plain type)
Substrate composition & dominance hierarchy	Pebble > Sand = Mud = Cobble	Sand > Gravel > Mud > Pebble = Cobble
Primary reference	Ko et al. (2013) [24]	Lee et al. (2010) [30]

Table S2. Statutory water quality standards and parameter thresholds for rivers and streams (living environment) stipulated by the Framework Act on Environmental Policy of the Republic of Korea. The environmental classifications range from Grade Ia (Very Good) to Grade VI (Very Poor). (BOD: biochemical oxygen demand, COD: chemical oxygen demand, DO: dissolved oxygen, SS: suspended solids, TN: total nitrogen, TP: total phosphorus, Total_Coli: total coliforms, Fecal_Coli: fecal coliforms)

Grade	Status	BOD (mg/L)	COD* (mg/L)	DO (mg/L)	SS (mg/L)	TN** (mg/L)	TP (mg/L)	Total_Coli (MPN/100mL)	Fecal_Coli (MPN/100mL)
**Ia**	**Very Good**	≤ 1	≤ 2	≥ 7.5	≤ 25	≤ 0.2	≤ 0.02	≤ 50	≤ 10
**Ib**	**Good**	≤ 2	≤ 4	≥ 5.0	≤ 25	≤ 0.3	≤ 0.04	≤ 500	≤ 100
**II**	**Slightly Good**	≤ 3	≤ 5	≥ 5.0	≤ 25	≤ 0.4	≤ 0.1	≤ 1,000	≤ 200
**III**	**Fair**	≤ 5	≤ 7	≥ 5.0	≤ 25	≤ 0.6	≤ 0.2	≤ 5,000	≤ 1,000
**IV**	**Slightly Poor**	≤ 8	≤ 9	≥ 2.0	≤ 100	≤ 1.0	≤ 0.3	-	-
**V**	**Poor**	≤ 10	≤ 11	≥ 2.0	-	≤ 1.5	≤ 0.5	-	-
**VI**	**Very Poor**	> 10	≤ 11	< 2.0	-	≥ 1.5	≥ 0.5	-	-

* The environmental grades for COD were determined based on the historical river water quality standards of the Ministry of Environment, Republic of Korea (prior to the national transition to Total Organic Carbon [TOC]).

** The TN environmental grades were evaluated based on the National Environmental Standards for Lakes and Reservoirs (Ministry of Environment, Republic of Korea).

Table S3. Quantitative physical metrics, microhabitat dimensions, standardized sampling gear specifications, and summary of the field-derived morphometric dataset for *Squalidus multimaculatus* in Baebong Stream, Goseong (April–September 2014). The structured operational matrix delineates fine-scale channel profiles and microhabitat-specific sample distribution (n = 676) stratified by depositional and erosional stream zones to support population demographic modeling.

Sampling attribute / Parameter	Deep pool zones	Shallow riffles and runs
Analyzed reach	Lower reaches / downstream	Lower reaches / downstream
River width (m)	80–100	2–20
Water width (m)	50–80	1.5–20
Water depth (m)	0.2–2.0	0.1–1.5
Substrate composition & dominance hierarchy	Mud > Pebble = Cobble > Sand > Gravel	Pebble > Mud > Gravel > Sand = Cobble
Deployed sampling gear	Cast net	Kick net
Mesh size specification	5 × 5 mm	4 × 4 mm
Standardized effort per site	2 hours	2 hours
Field-derived dataset type	Microhabitat-specific morphometric dataset	
Measured variable	Total length and body weight	
Individuals	n = 526	n = 150
Total length range	26.60–104.39 mm TL	56.00–99.80 mm TL
Weight range	0.1–12.3 g	2.1–10.3 g

Table S4. Long-term monotonic trend analysis of monthly water quality parameters (1997–2025; Seasonal Mann–Kendall test) and annual aquatic thermal indices (Standard Mann–Kendall test) in the newly colonized Goseong coastal stream habitat.

Parameter	Method	Kendall's τ	Sen's Slope	p-value	Monotonic Trend
**BOD**	Seasonal Mann-Kendall	0.0799	0.0000	0.0343	Stable
**COD**	Seasonal Mann-Kendall	0.2896	0.0389	< 0.0001	Increase
**DO**	Seasonal Mann-Kendall	0.1381	0.0250	0.0003	Increase
**SS**	Seasonal Mann-Kendall	-0.0718	-0.0111	0.0642	No Trend
**TN**	Seasonal Mann-Kendall	0.0196	0.0008	0.6086	No Trend
**TP**	Seasonal Mann-Kendall	0.2072	0.0003	< 0.0001	Increase (functionally stable)
**Total_Coli**	Seasonal Mann-Kendall	-0.1560	-16.8615	< 0.0001	Decline
**Fecal_Coli**	Seasonal Mann-Kendall	0.0000	0.0000	0.9807	No Trend
**Wtemp**	Seasonal Mann-Kendall	0.1107	0.0667	0.0039	Increase
**Mean_Wtemp**	Standard Mann–Kendall	0.2980	0.0487	0.0244	Increase
**Min_Wtemp**	Standard Mann–Kendall	0.4433	0.1191	0.0007	Sharp Increase
**Max_Wtemp**	Standard Mann–Kendall	0.0222	0.0000	0.8801	No Trend

Table S5. Root-mean-square error (RMSE) values for monthly mean air and water temperatures, comparing the native baseline to the three temporal phases of the colonized habitat. For air temperature, the extended baseline (Y-T_0^*) was used for comparison against the Goseong phases (G-T_0^*, G-T_1, and G-T_2). For water temperature, the standard baseline (Y-T_0) was used for comparison against the corresponding Goseong phases (G-T_0, G-T_1, and G-T_2).

Parameter	Comparison	RMSE
**Air temperature**	G-T_0^* vs. Y-T_0^*	2.58
	G-T_1 vs. Y-T_0^*	1.63
	G-T_2 vs. Y-T_0^*	1.23
**Water temperature**	G-T_0 vs. Y-T_0	2.52
	G-T_1 vs. Y-T_0	2.38
	G-T_2 vs. Y-T_0	1.66

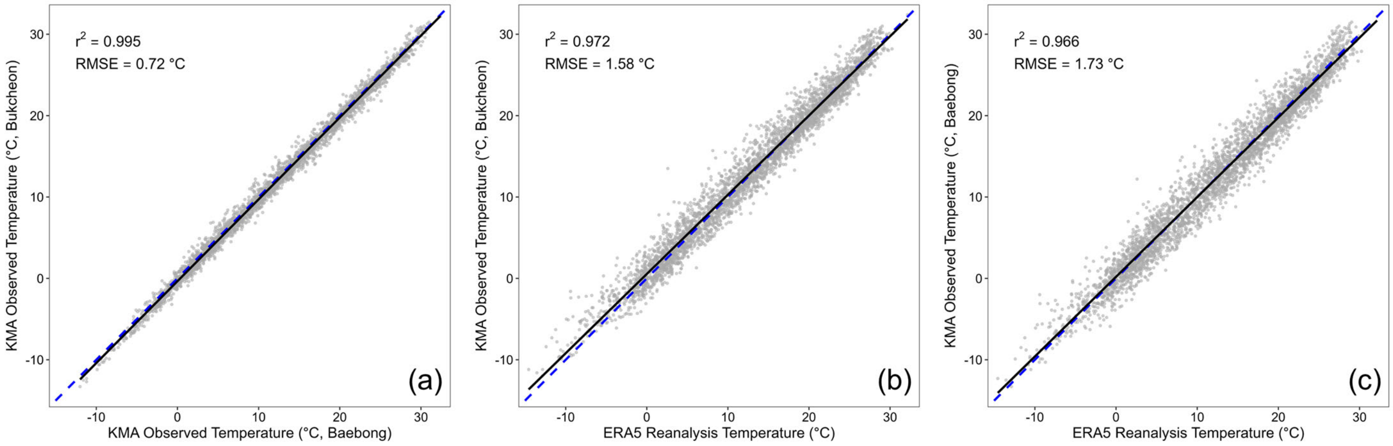

Figure S1. Comprehensive cross-validation of daily mean air temperatures across local ground observation stations and the ERA5 reanalysis dataset (2016–2025). (a) Direct empirical comparison between the Korea Meteorological Administration (KMA) automated weather stations at Baebong Stream and Bukcheon Stream (r^2 = 0.995, RMSE = 0.72 °C, $p < 0.001$). (b) Validation of the ERA5 reanalysis product against Bukcheon Stream KMA ground observations (r^2 = 0.972, RMSE = 1.58 °C, $p < 0.001$). (c) Validation of the ERA5 reanalysis product against Baebong Stream KMA ground observations (r^2 = 0.966, RMSE = 1.73 °C, $p < 0.001$). The solid black lines represent the linear regression fits, and the blue dashed lines indicate the 1:1 reference line.
